# Supplementary material for: Performance of formal smell testing and symptom screening for identifying SARS-CoV-2 infection
Source: PLoS One. 2022 Apr 12;17(4):e0266912. doi: 10.1371/journal.pone.0266912 (PMC9004758; doi:10.1371/journal.pone.0266912)
Supplement: S1 Table — (DOCX) [file pone.0266912.s001.docx]

Supplemental Table S1. Demographics, health history, symptoms, and SARS-CoV-2 status by smell testing performance

|  | Hyposmia  (PST≤5; N=83) | | Normosmia  (PST 6-8; N=221) | |  |
| --- | --- | --- | --- | --- | --- |
| Characteristic | n | % | n | % | *P* |
| Demographic |  |  |  |  |  |
| Age (years; mean, SD) | 48.9 | 19.62 | 43.9 | 16.90 | 0.03 |
| Female | 41 | 49.4% | 120 | 53.3% | 0.58 |
| Race |  |  |  |  | 0.83 |
| Asian | 1 | 1.2% | 1 | 0.5% |  |
| Black | 13 | 15.7% | 33 | 14.9% |  |
| White | 68 | 81.9% | 185 | 83.7% |  |
| Multiracial | 0 | 0.0% | 1 | 0.5% |  |
| Hispanic | 3 | 3.6% | 7 | 3.2% | 0.94 |
| Health history |  |  |  |  |  |
| Loss of smell | 15 | 18.1% | 14 | 6.3% | 0.002 |
| Nasal trauma | 12 | 14.5% | 36 | 16.3% | 0.70 |
| Nasal polyps | 2 | 2.4% | 9 | 4.1% | 0.49 |
| Nasal sinus surgery | 7 | 8.4% | 13 | 5.9% | 0.42 |
| Other nasal surgery | 5 | 6.0% | 13 | 5.9% | 0.96 |
| Oxygen use at home by nasal cannula | 1 | 1.2% | 7 | 3.2% | 0.34 |
| Difficulty breathing through nose daily | 4 | 4.8% | 13 | 5.9% | 0.72 |
| Daily nasal drainage | 10 | 12.0% | 28 | 12.7% | 0.88 |
| Nasal drainage after meals | 4 | 4.8% | 8 | 3.6% | 0.63 |
| Use prescription nasal sprays regularly | 13 | 15.7% | 10 | 4.5% | 0.001 |
| Use over the counter nasal steroid sprays regularly | 17 | 20.5% | 19 | 8.6% | 0.004 |
| Use other over the counter nasal sprays regularly | 13 | 15.7% | 15 | 6.8% | 0.02 |
| Use of any nasal spray regularly | 23 | 27.7% | 34 | 15.4% | 0.01 |
| Ever taken zinc-containing medications or cold remedies | 2 | 2.4% | 8 | 3.6% | 0.60 |
| Recent (past 2 weeks) cold affecting nasal breathing | 5 | 6.0% | 19 | 8.6% | 0.46 |
| Seasonal allergies | 44 | 53.0% | 117 | 52.9% | 0.99 |
| Recurrent or chronic sinus infections | 17 | 20.5% | 31 | 14.0% | 0.17 |
| Deviated nasal septum | 2 | 2.4% | 8 | 3.6% | 0.60 |
| Nasal polyps | 4 | 4.8% | 9 | 4.1% | 0.77 |
| Parkinson’s disease | 2 | 2.4% | 2 | 0.9% | 0.31 |
| Dementia | 1 | 1.2% | 1 | 0.5% | 0.47 |
| Symptom/exposure screening |  |  |  |  |  |
| Previous positive SARS-CoV-2 test or exposure to someone with COVID-19 | 15 | 18.1% | 13 | 5.9% | 0.001 |
| Reported fever | 10 | 12.0% | 17 | 7.7% | 0.23 |
| Shortness of breath | 14 | 16.9% | 30 | 13.6% | 0.47 |
| Cough | 16 | 19.3% | 30 | 13.6% | 0.22 |
| Chills | 10 | 12.0% | 18 | 8.1% | 0.29 |
| Sore throat | 7 | 8.4% | 16 | 7.2% | 0.73 |
| Loss of taste/smell | 9 | 10.8% | 5 | 2.3% | 0.001 |
| Body aches | 12 | 14.5% | 22 | 10.0% | 0.27 |
| Maximum recorded temperature in emergency department (mean, SD in °F) | 98.7 | 1.17 | 98.5 | 0.96 | 0.37 |
| Recorded fever in ED (T >= 100.4 °F (38 °C)) | 6 | 7.2% | 9 | 4.1% | 0.26 |
| SARS-CoV-2 positive | 47 | 56.6% | 41 | 18.6% | <0.001 |

ED, emergency department; PST, pocket smell test; SD, standard deviation
