# Supplementary material for: Performance of formal smell testing and symptom screening for identifying SARS-CoV-2 infection
Source: PLoS One. 2022 Apr 12;17(4):e0266912. doi: 10.1371/journal.pone.0266912 (PMC9004758; doi:10.1371/journal.pone.0266912)

Supplemental Figure S1. Receiver operating characteristic (ROC) curves for identifying SARS-CoV-2 infection for each of the eight tested odors


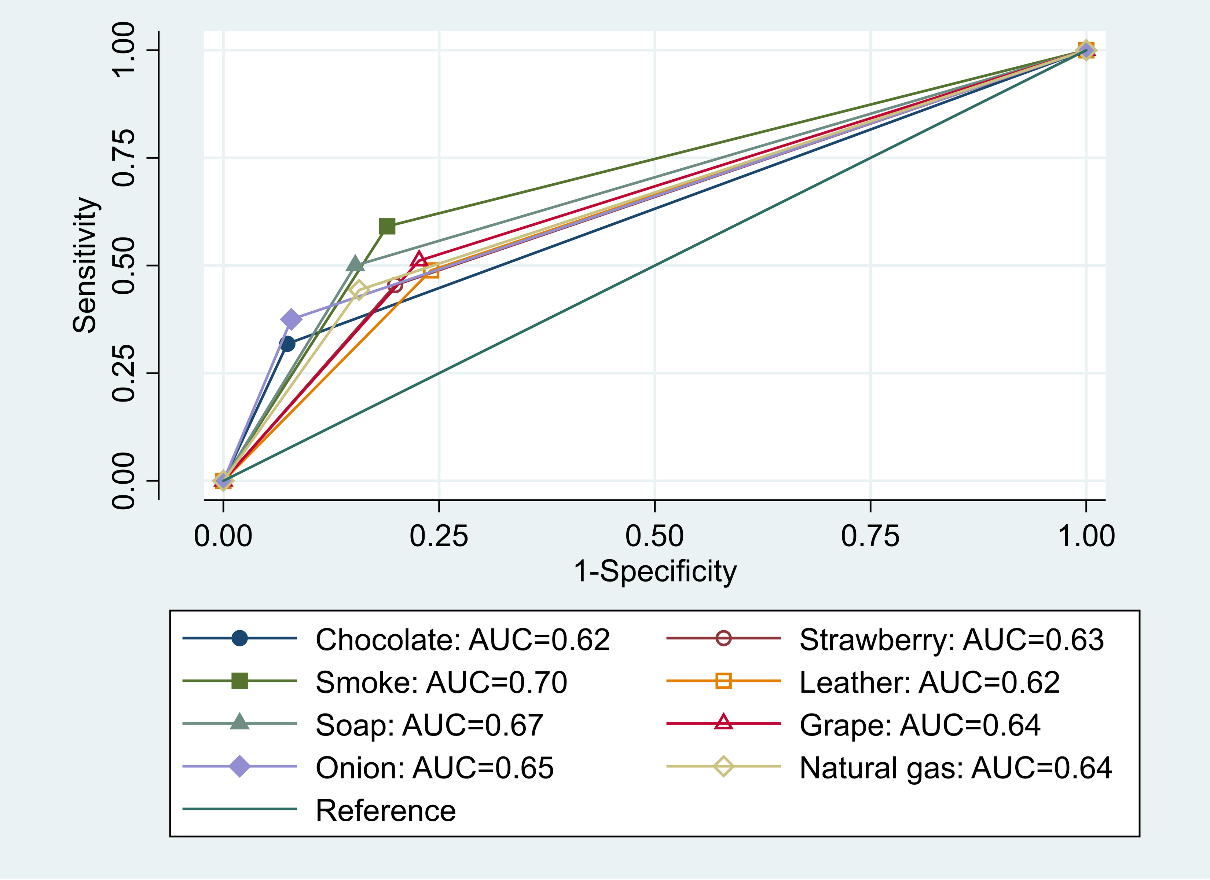

Supplement: S1 Fig — (DOCX) [file pone.0266912.s002.docx]
