# Supplementary material for: Performance of formal smell testing and symptom screening for identifying SARS-CoV-2 infection
Source: PLoS One. 2022 Apr 12;17(4):e0266912. doi: 10.1371/journal.pone.0266912 (PMC9004758; doi:10.1371/journal.pone.0266912)
Supplement: Attachment — Submitted filename: Review report smell.docx [file pone.0266912.s004.docx]

# Review comments

*The authors have an insightful work that points towards the use of olfactory assessment as an important tool in the screening (and diagnostic utility) for COVID-19. The paper capitalized that, being non-invasive and less stressful, the smell test (PCT) is a convenient method that can be employed in diagnosing COVID-19, particularly important in community settings with resource limitations. In general, the manuscript is well written, and it should be considered for publication provided that the suggested modifications both from the reviewer and academic editor(s) are taken into account. Below are the main comments of the review report.

*There are some ambiguous sentences in the abstract section. For example: “…Twenty-eight had a SARS-CoV-2 positive 49 patients (31.8%) …” And the abstract should be clearer, and if possible, fascinating with parsimony.

* Eligibility criteria are not clearly elucidated; inclusion and exclusion criteria should be detailed. This is particularly important as the study focused in patients visiting the emergency department.

* The statistical analyses performed are described in sufficient detail, and conclusions are presented in an appropriate fashion and are supported by the data.

* In the limitation section/discussion part, some points should be raised, including: use of this olfaction-based screening should be deferred in those with reported baseline smell problems or smell loss (diminution).

*There are some punctuation errors. For instance, in-text citations were written after punctuation marks. The numbers should precede the punctuation.

*There is no a separate list of abbreviations/acronyms in the paper. But, if it is part of the journal structure, it is acceptable.

*Regarding plagiarism, I trust the academic editors will analyze the similarity index of the document, as a routine part of the decision making process.
