# Supplementary material for: Performance of formal smell testing and symptom screening for identifying SARS-CoV-2 infection
Source: PLoS One. 2022 Apr 12;17(4):e0266912. doi: 10.1371/journal.pone.0266912 (PMC9004758; doi:10.1371/journal.pone.0266912)
Supplement: Attachment — Submitted filename: Response to reviewers #2.docx [file pone.0266912.s006.docx]

March 16, 2022

Dear Editor and Reviewers,

Thank you for your many helpful comments and questions regarding our manuscript titled *Performance of formal smell testing and symptom screening for identifying SARS-CoV-2 infection*.

We placed your comments into a table with our responses adjacent. When appropriate we indicate the line numbers where we revised the manuscript.

Regards,

James W Keck, MD MPH

Assistant Professor

Department of Family and Community Medicine

University of Kentucky

| **Comments to the Author** | **Authors’ Responses** |
| --- | --- |
| **Reviewer #1** |  |
| Line 125: Figure 1 -- caption meant to be here in the methods section? | Yes – per PLOS One author guidelines the figure caption should be placed after the paragraph where it is first reference. Figure 1 is referenced in the preceding paragraph. |
| Lines 131-132: understood but not quite grammatically clear | Removed clause “based on unique medical record number” to improve sentence syntax. |
| Lines 152: understood perhaps specifying what "best performance" means | Clarified that the performance metric we used was AUC (area under the curve) |
| Line 234: interesting perhaps unexpected given that nasal steroids did impact performance, correct? And assuming most people with rhinitis on nasal steroids? | Looking at S1 Table, about 50% of sample reported seasonal allergies (believable in Kentucky). However, only 12% reported nasal steroid use. We modified this sentence (lines 234-36) to represent our analysis more accurately, which found that smell testing performance was similar between patients with/without sinusitis and seasonal allergies. We didn’t measure whether either condition was an effect modifier of the discriminatory ability of smell testing for SARS-CoV-2 infection. |
| Line 276: or negative PCR and sustained olfactory dysfunction that would decrease the already high specificity, yes? but no need to specifically note to avoid wordage. | We agree that this scenario is also possible and would decrease the specificity of the smell test. |
| **Reviewer #3** |  |
| Discussion: There is some ambiguity in line (≠250 and ≠251): …the utility of this using this as..? | We revised the sentence to read “*The performance of smell testing in asymptomatic patients suggests its utility as a SARS-CoV-2 screening tool in asymptomatic populations, like employees at congregate work settings”* for clarity. |
| **Reviewer #6** |  |
| The quality of this manuscript will be improved if more information is added to the background to the abstract as well as the conclusion of the abstract. | We minimally revised the background section of the abstract for improved clarity.  Respectfully, we are unclear on what additional information would be helpful in the abstract. An abstract provides a concise description of the study and invites the reader to review the full manuscript for greater detail. As PLOS One is open access, anyone can read the manuscript in its entirety. |
